# Analyzing the Effect of Dried Shrimp on the Flavor of Sheep Bone Soup Through Sensory Evaluation Combined with Untargeted Approaches

**DOI:** 10.3390/foods14081425

**Published:** 2025-04-21

**Authors:** Qiuyu Zhu, Lili Zhang, Xingming Sun, Baoguo Sun, Yuyu Zhang

**Affiliations:** 1 Key Laboratory of Geriatric Nutrition and Health, Beijing Technology and Business University, Ministry of Education, Beijing 100048, China; zhuqiuyu0821@163.com (Q.Z.); zhanglili921116@163.com (L.Z.); 13676972186@163.com (X.S.); sunbg@btbu.edu.cn (B.S.); 2 Beijing Life Science Academy, Beijing 102209, China; 3 Food Laboratory of Zhongyuan, Beijing Technology and Business University, Beijing 100048, China; 4 Key Laboratory of Flavor Science of China General Chamber of Commerce, Beijing Technology and Business University, Beijing 100048, China

**Keywords:** sheep bone soup, dried shrimp, stewing process, orthogonal experiment, taste compounds

## Abstract

Dried shrimp is a popular dietary ingredient that is often included in appetizer soups, stir-fry dishes, or other stews to improve the umami taste. The effects of adding dried shrimp on the sensory characteristics and taste components of sheep bone soup were investigated through sensory evaluation and untargeted approaches. The results of the single-factor and orthogonal experiments showed that the flavor qualities of sheep bone soup were optimal under the following conditions: 30% dried shrimp added, a 1:4.5 material–water ratio, and 2.7 h of stewing time. Sensory analysis showed a significant increase in the aroma, umami, kokumi, and texture intensity of the optimized sheep bone soup with dried shrimp. The untargeted approach combined with multivariate statistical analysis showed that compounds with a sweet taste (Lys and Ser), a umami taste and umami enhancement (Ala-Leu, Glu-Pro, Glu-Glu, Asp-Phe, pyroglutamic acid, and cinnamic acid), a bitter taste (Gly-Leu, Leu-Leu, Ile-Lys, and taurine), a kokumi taste (γ-Glu-Met, γ-Glu-Leu, γ-Glu-Ile, N-acetylmethionine, and N-acetylphenylalanine), a sour taste (malic acid), and a popcorn-like aroma (2-acetylthiazole) contributed significantly to the flavor enhancement of sheep bone soup. In addition, the contribution of Ac-Ser-Asp-Lys-Pro could not be ignored. These results contribute to a better understanding and improvement of the flavor qualities of sheep bone soup.

## 1. Introduction

Sheep are one of the major livestock breeds for meat production globally, with byproducts such as bones, cartilage, and carcass trimmings accounting for about 10–30% of their live weight [1]. Due to the rapidly increasing production of sheep in recent years, slaughtering produces a large amount of bones [2,3]. It is estimated that approximately 20 million tons of sheep bones are produced annually in China [4]. These bones are an excellent source of high-quality proteins, fatty acids, minerals, and other essential nutrients [5]. However, only a small portion of these bones are utilized as animal feed, which has low market value. The majority of the bones end up in landfills, burdening the environment and resulting in a waste of natural resources [6].

Sheep bone soup is a dish made by stewing sheep bones as the raw material, and it is consumed globally due to its unique nutritional properties, physicochemical composition, sensory attributes, and distinct flavors [7]. The distinct taste of the soup comes from protein degradation, lipid oxidation, and the Maillard reaction, all of which result in the dissolution of volatile and nonvolatile compounds into the soup [8]. However, sheep bone soup faces problems such as the lack of variety in the use of a single raw material, insufficient taste, and poor texture, resulting in sheep bone soup products rarely being sold on the market. Thus, it is necessary to develop cooking technologies to preserve the nutrients and flavor of sheep bone soup. Current research on enhancing the release of nutrients and flavor formation in bone soup primarily focuses on three main aspects: pre-processing, stewing conditions, and additive formulas. Pre-processing (such as pre-cooking and enzymatic hydrolysis) and optimizing basic cooking parameters (such as stewing temperature and stewing time) can better retain the original flavor of the bone soup [9,10,11,12]. Adding exogenous substances like mushrooms and Welsh onions can increase the efficiency and simplicity of the cooking process while improving the diversity of the flavor, taste, and related substances of the bone soup [13,14].

Combining different animal products in stews is a classic and widely used practice in the cooking process, especially for fish and sheep dishes [15,16]. Dried shrimp, produced by boiling and drying *Acetes chinesis*, is one of the most widely consumed marine products. It has a delicious taste, is low in fat, and is rich in nutrients (mainly protein and minerals) as well as in bioactive peptides with antioxidant, antihypertensive, and immune-enhancing properties [17]. Dried shrimp is commonly used as a flavor enhancer, due to its richness in amino acids with umami (Glu and Asp) and sweet (Thr, Ser, Gly, etc.) flavors [18]. Liu et al. found that the addition of dried shrimp enzymatic hydrolysate to wine increased the content of the taste compounds and improved the overall taste of the wine [19]. Sun et al. found that the addition of shrimp could enhance the taste, inhibit the saltiness, and improve the texture of compound seasoning [20]. However, few studies have been conducted on the effect of dried shrimp on the sensory characteristics of sheep bone soup, limiting the widespread consumption of this soup to some extent. The untargeted approach is a widely used method in studying the flavor of products, allowing for the simultaneous detection of more than a dozen or even hundreds of endogenous chemical compounds [21]. Qualitative and quantitative analyses of taste compounds provide a basis of data for assessing changes in the taste of sheep bone soup.

In this work, the effects of dried shrimp addition on the sensory characteristics and taste compounds of sheep bone soup were explored. Based on a single-factor experiment, an orthogonal experiment was designed to optimize the process conditions. Furthermore, the changes in taste compounds in sheep bone soup before and after adding dried shrimp were determined using the untargeted approach via UHPLC-QE-MS. This work contributes to a better understanding of the flavor enhancement of sheep bone soup with dried shrimp and the development of high-quality sheep bone soup products.

## 2. Materials and Methods

### 2.1. Materials

The sheep bones were purchased from Shenzhou Food Group Co. (Heze, China); dried shrimp were purchased from the local market in Taizhou, China; ultrapure water was supplied by Watsons (Guangzhou, China); chromatography-grade methanol and HPLC-grade methanoic acid were obtained from Shanghai Aladdin Biochemical Technology Co., Ltd. (Shanghai, China); and chromatography-grade acetonitrile and acetic acid (≥99%) were procured from Merck (Darmstadt, Germany).

### 2.2. Preparation of Stewed Sheep Bone Soup with Dried Shrimp (SBS)

The SBS was processed according to the method described by Huang et al., with slight modifications [22]. In brief, the sheep bones were cut lengthwise into strips measuring 2 × 1 × 1 cm and stored at −20 °C. The sheep bones (50 g), water (200 mL), and dried shrimp (15 g) were mixed and stewed at 100 °C for 2 h. After stewing, the mixture was cooled to room temperature and then filtered through gauze to remove any solids. The filtrates were used for sensory evaluation, and the sheep bone soup without dried shrimp was prepared in order to serve as a blank control (CK).

### 2.3. Optimization of the Stewing Conditions

First, the effects on the flavor of SBS when changing a single factor were investigated. Three factors were examined: the added content of dried shrimp, stewing time, and material–water ratio. The experimental design for the single-factor test is listed in Table S1 (Supplementary Materials). Subsequently, the stewing parameters were further optimized using three-factor, three-level orthogonal tests. The levels of the three factors were set based on the results of the single-factor tests. The experimental design for the orthogonal tests is listed in Table S2 (Supplementary Materials).

### 2.4. Sensory Evaluation of SBS

All sensory evaluation experiments were approved by the Ethics Committee of Beijing Technology and Business University (No. 2023056, Beijing, China). Prior to the sensory evaluation, 10 panelists (5 men and 5 women; 23–34 years old) signed an informed consent form and were informed of the details of the experiment. These panelists were also required to undergo sensory evaluation training for 3 weeks (twice a week, with each training session lasting 1 h). Samples, with their temperatures ranging from 50 to 60 °C, were randomly distributed to the panelists. All participants rated the samples based on color (X_1_), aroma (X_2_), umami (X_3_), kokumi (X_4_), texture (X_5_), and overall impression (X_6_), following the sensory evaluation criteria outlined in Table S3, and the final score of the samples (X) was calculated according to the following formula:

X = 0.1X_1_ + 0.3X_2_ + 0.4X_3_ + 0.1X_4_ + 0.1X_5_ + 0.3X_6_

### 2.5. Untargeted Analysis

#### 2.5.1. Extraction of Compounds

The extraction procedure was based on that of Chen et al. with some modifications [23]. During extraction, 100 mg of each sample was combined with 400 μL of pre-cooled liquid extractant (methanol/acetonitrile = 3:1) and vortexed (TYXH-I, Hanuo Instruments, Shanghai, China) for 5 min. Initially, the mixture was treated with ultrasound (F-060SD, Fuyang Technology, Shenzhen, China) at 80 W for 15 min and then maintained at 4 °C for 2 h. Subsequently, the mixture was centrifuged (TGL-16MS, Bioridge Centrifuge Instruments, Shanghai, China) for 15 min (12,000× *g*, 4 °C). The resulting supernatant was collected and dried in a freeze-dryer (LNG-T98, Huamei Biochemical Instruments, Taicang, China). The lyophilized product was reconstituted by adding 100 μL of 50% methanol solution and being vortexed for 3 min (2000× *g*, 4 °C). The samples were then centrifuged at 12,000× *g* for 15 min at 4 °C, and the supernatant was used for compound analysis. Quality control (QC) samples were prepared by mixing equal amount (200 μL) of each sample.

#### 2.5.2. Ultra-High-Performance Liquid Chromatography/Q Exactive HF-X Hybrid Quadrupole–Orbitrap Mass Spectrometer (UHPLC-QE-MS) Analysis

UHPLC-QE-MS analysis was performed using a UHPLC system (Vanquish, Thermo Fisher Scientific, Waltham, MA, USA) equipped with an ACQUITY UPLC HSS T3 column (150 mm × 2.1 mm × 1.8 μm, Waters, Wilmslow, UK). The system was coupled to a Q Exactive HF-X mass spectrometer (Orbitrap MS, Thermo Fisher Scientific, Waltham, MA, USA) operating in both positive and negative modes. The parameters were set as follows: a 2 μL injection volume, 40 °C column temperature, and 0.3 mL/min mobile-phase flow. For the positive mode, the mobile phase involved 0.1% methanoic acid in water (A) and methanol (B). An increasing linear gradient of solvent B (*v*/*v*) was used as follows: 0–0.5 min, 2% B; 0.5–6 min, 2~50% B; 6–10 min, 50~98% B; 10–14 min, 98% B; 14–16 min, 98~2% B; and 16–21 min, 2% B. For the negative mode, the mobile phase involved 0.05% acetic acid in water (C) and methanol (D). An increasing linear gradient of solvent D (*v*/*v*) was used as follows: 0–0.5 min, 2% D; 0.5–6 min, 2~50% D; 6–10 min, 50~98% D; 10–14 min, 98% D; 14–16 min, 98~2% D; and 16–21 min, 2% D. To ensure sequence analysis stability throughout the analyses, QC samples were acquired after every 6 samples.

The QE HF-X mass spectrometer operated in both positive and negative ion modes, using the following parameters: the ESI-MS^n^ was used with a spray voltage of 2.5 kV; the collision energy was 30 V; sheath gas and auxiliary gas were set at 30 and 10 arbitrary units, respectively; and the capillary temperature was 325 °C. The analyzer scanned over a mass range of *m*/*z* 70-1050 for a full scan. The QE HF-X mass spectrometer was used for acquiring full-scan MS/MS spectra in information-dependent acquisition (IDA) mode under software (Xcalibur, Thermo Fisher Scientific, Waltham, MA, USA) control.

#### 2.5.3. Data Analysis

The raw data were first converted to the mzXML format using ProteoWizard software (Version 3.0.21229). Then, the data were processed via R package analysis to perform baseline filtering, peak recognition, integration, peak alignment, and retention time. The characteristic peaks in QC samples with a relative standard deviation (RSD) > 30% were filtered to obtain the data matrix of retention time (RT), the mass-to-charge ratio (*m*/*z*), and peak intensity. The identification of the compounds was confirmed as follows: accuracy *m*/*z* values (<25 ppm) and the fragment information obtained according to the MS/MS pattern was compared with the Human Metabolome Database (HMDB) (http://www.hmdb.ca, accessed on 2 July 2024), MassBank (http://www.massbank.JP/, accessed on 2 July 2024), and mzCloud (https://www.mzcloud.org, accessed on 2 July 2024). To reduce the error caused by the sample preparation process and the instrument, peak area intensities were normalized to the total spectral intensity, and the data matrix for subsequent analysis was obtained. Multivariate analyses were carried out using the OmicShare cloud platform (https://www.omicshare.com, accessed on 30 July 2024).

### 2.6. Statistical Analysis

Data were expressed as the mean ± standard deviation (n = 3). One-way analyses of variance (ANOVAs) and Duncan’s multiple tests were conducted using IBM SPSS Statistical 26.0 software to determine significant differences among the mean values at *p* < 0.05. Graphs were plotted using Origin 2024 software.

## 3. Results and Discussion

### 3.1. Optimization of Stewing Conditions

#### 3.1.1. Effect of Content of Dried Shrimp Addition on Sensory Quality of SBS

**Figure 1:**
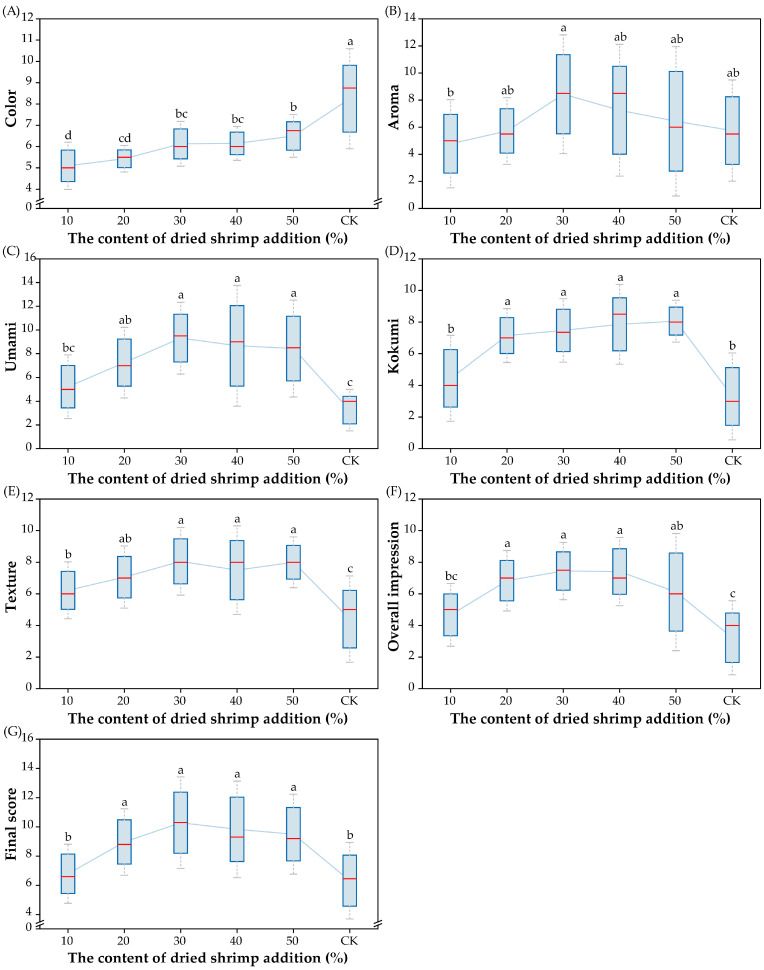
Effect of content of dried shrimp addition on sensory quality of SBS ((**A**) score for color; (**B**) score for aroma; (**C**) score for umami; (**D**) score for kokumi; (**E**) score for texture; (**F**) score for overall impression; (**G**) final score). Significant differences between soup samples are represented by different lowercase letters (n = 10) (*p* < 0.05).

The effect of the addition of different dried shrimp contents (10%, 20%, 30%, 40%, and 50%; percentage of total weight of sheep bones) on the sensory quality of SBS was studied when stewing times and material–water ratios were fixed. As shown in Figure 1, the score of the CK group was higher than those of the other five groups in terms of color. As the cooking time extended, the color of CK gradually changed from transparent to white in this study, indicating the emulsification of the soup. The addition of dried shrimp resulted in a change in the color of the SBS to yellow, which may have been a result of the release of pigments from the dried shrimp during the stewing process [24]. Adding dried shrimp also increased the aroma score of the SBS. The highest aroma score (8.5) was obtained when 30% of dried shrimp was added, and thereafter, the aroma score was negatively correlated with higher content addition. This meant that the appropriate addition of dried shrimp could inhibit or cover the off flavor present in sheep bone soup. However, excessive addition aggravated the off flavor of the sheep bone soup due to the distinctive odor of dried shrimp. In addition, the umami score of the SBS significantly increased after adding dried shrimp (*p* < 0.05), which might have been caused by the release of amino acids, minerals, and other components in the shrimp during the stewing process. Moreover, the dried shrimp addition improved the texture and kokumi score of the soup. There was no significant difference in the texture and kokumi score when dried shrimp was added at contents of 20%~50% (*p* > 0.05). The overall impression score of the SBS with 30% and 40% dried shrimp added was higher than those of the other three groups, and the final score revealed that sheep bone soup with 30% dried shrimp added was the most popular with the highest sensory evaluation score of 10.3. These findings indicate that adding dried shrimp is an effective way to improve the overall flavor of sheep bone soup, particularly in terms of its aroma and umami profile. Despite the overall impression score of SBS being the highest with 30% dried shrimp added, it was not very desirable for the individuals in this study. Hence, further optimization of the stewing conditions is essential to enhance the sensory profile of the final product.

#### 3.1.2. Effect of Stewing Time on Sensory Quality of SBS

**Figure 2:**
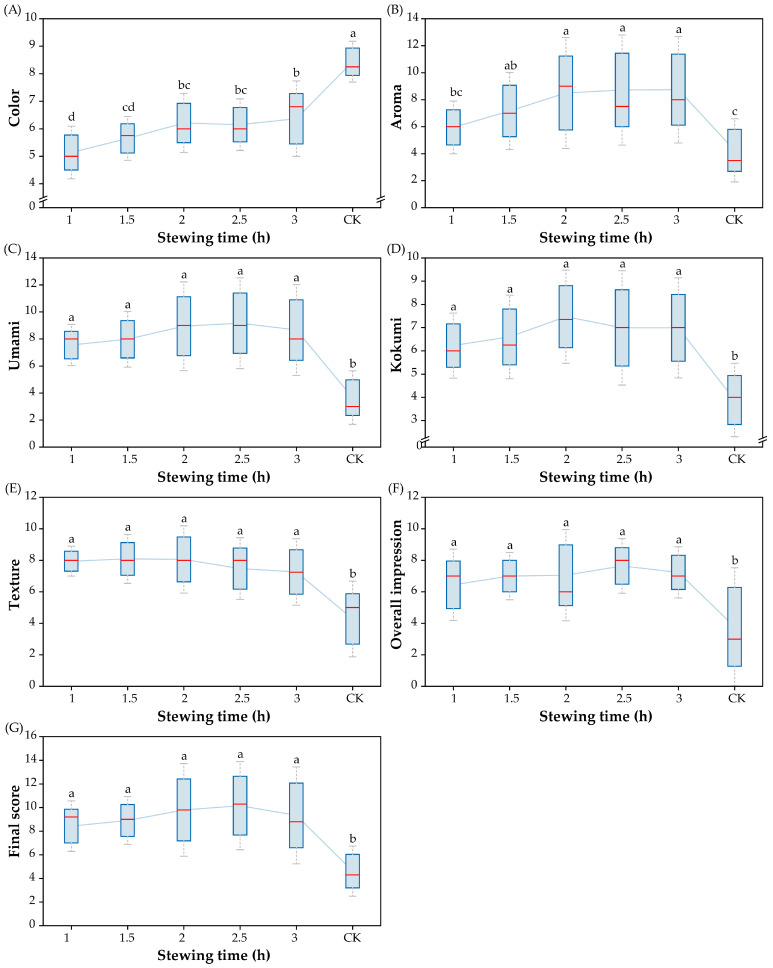
Effect of stewing time on sensory quality of SBS ((**A**) score for color; (**B**) score for aroma; (**C**) score for umami; (**D**) score for kokumi; (**E**) score for texture; (**F**) score for overall impression; (**G**) final score). Significant differences between soup samples are represented by different lowercase letters (n = 10) (*p* < 0.05).

Stewing time is an important factor in the dissolution and release of macromolecules (such as proteins and fats) from the sheep bone. The effect of stewing time (1 h, 1.5 h, 2 h, 2.5 h, and 3 h) on the sensory quality of SBS was investigated when the content of dried shrimp addition and material–water ratio were fixed. As shown in Figure 2, the color score of SBS gradually increased with longer stewing times, and when stewed for 3 h, the color score reached the maximum. A significant increase was observed in the aroma score with increasing stewing time from 1 h to 2 h. This upward trend can be explained by the fact that continuous heating caused further oxidation of the lipids [25]. After stewing for 2.5 h, the aroma score of SBS tended towards stability. A similar trend could be observed in the kokumi score. Guan et al. pointed out that the flavor composition variation stabilized after stewing for 4 h, and the sensory evaluation results also showed that the umami, saltiness, and aroma scores of chicken soup were no longer significantly increased [26]. The scores of texture (7.9–8.1) increased slightly at a stewing time of 1–2 h and decreased with longer stewing times. The scores for umami, kokumi, texture, and overall impression had no significant difference among the SBS samples at different stewing times. However, the soup stewed for 2.5 h showed the highest umami, overall impression, and final scores, which meant that a stewing time of 2.5 h was appropriate for the preparation of SBS. These findings are similar to the results obtained for dark meat when cooking it for 2.5 h [27].

#### 3.1.3. Effect of Material–Water Ratio on Sensory Quality of SBS

**Figure 3:**
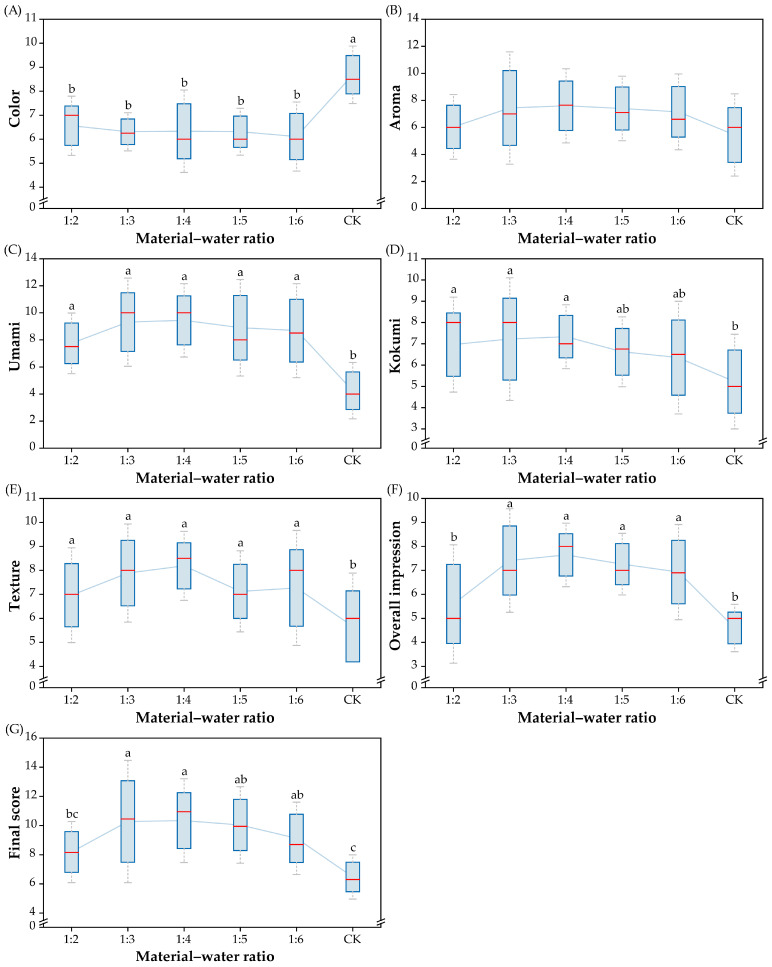
Effect of material–water ratios on sensory quality of SBS ((**A**) score for color; (**B**) score for aroma; (**C**) score for umami; (**D**) score for kokumi; (**E**) score for texture; (**F**) score for overall impression; (**G**) final score). Significant differences between soup samples are represented by different lowercase letters (n = 10) (*p* < 0.05).

The effect of different material–water ratios (1:2, 1:3, 1:4, 1:5, and 1:6) on the sensory quality of SBS was investigated when the content of dried shrimp addition and stewing times were fixed. As shown in Figure 3, the color score of the SBS decreased with increasing ratios from 1:2 to 1:4. The umami, kokumi, and texture scores of the SBS were noticeably higher than those of the CK group (*p* < 0.05); however, there was no significant difference in these scores when the material–water ratio varied from 1:2 to 1:4. In addition, the results showed that the highest scores of aroma (7.6), umami (9.4), kokumi (7.3), and texture (8.2) were obtained at the material–water ratio of 1:4. The overall impression score of the SBS was significantly higher than that of the CK group (*p* < 0.05) (Figure 3). When the material–water ratio varied from 1:2 to 1:4, the overall impression score of the SBS rapidly increased and reached a peak of 7.6 at a ratio of 1:4. Then, the overall impression score decreased slightly when the ratio exceeded 1:6. The final score further confirmed that the sample stewed at ratio of 1:4 was the most popular. A favorable material–water ratio is important for the stewing process since the release of components is responsible for nutrition and flavor formation. Less water is not conducive to the release of nutritional components into SBS, and too much water could dilute the soup, which might explain the lower score of the SBS cooked at lower or higher material–water ratios. Similar results were also observed in a previous study by Liu et al. [28].

#### 3.1.4. Optimization of Stewing Conditions Using Orthogonal Tests

To determine the combined effect of the independent variable selected based on the results of the single-factor tests on the sensory quality, an orthogonal test with three independent variables at three levels was carried out. The results of the orthogonal tests are listed in Table 1, which shows that the order of significant factors for the sensory quality of the SBS samples was material–water ratio > content of dried shrimp addition > stewing time. Furthermore, based on the K values associated with the stewing parameters in the table, the sensory evaluation achieved the maximum scores when the content of dried shrimp added was 30%, the stewing time was 2.7 h, and the material–water ratio was 1:4.5.

### 3.2. Overview of Identified Compounds

**Figure 4:**
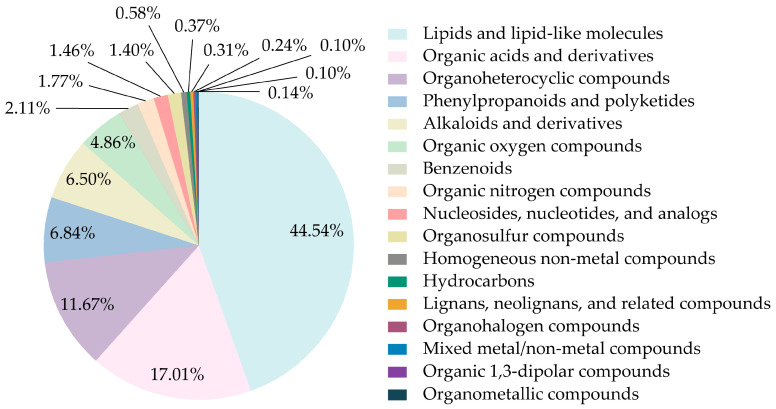
The composition and proportion of compounds identified by HMDB.

To investigate the change in chemical composition during stewing, both positive and negative ion modes were employed during UHPLC-QE-MS analysis based on additive ions, molecular ion peaks, and fragment ions using databases. A total of 5260 ion features of the compounds were extracted from all samples. Among them, 2939 compound features (55.87% of the total) were divided into 17 superclasses via comparisons with the HMDB (Figure 4). The top six superclasses were lipids and lipid-like molecules (1309 compounds, 44.54%), organic acids and their derivatives (500 compounds, 17.01%), organoheterocyclic compounds (343 compounds, 11.67%), organic oxygen compounds (201 compounds, 6.84%), benzenoids (191 compounds, 6.50%), and phenylpropanoids and polyketides (143 compounds, 4.86%).

### 3.3. Multivariate Statistical Analysis

**Figure 5:**
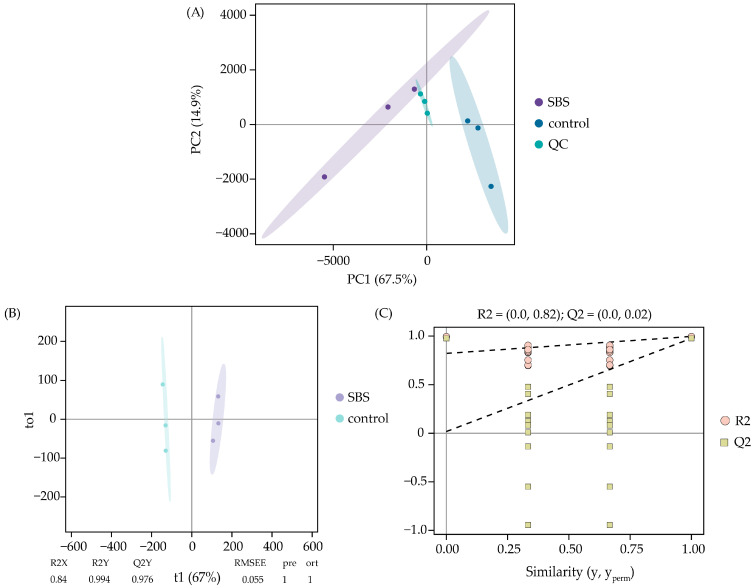
Multivariate analysis of chemical composition differences in sheep bone soup with or without dried shrimp ((**A**) score plot of PCA; (**B**) score plot of OPLS-DA; (**C**) response of 200 permutation tests for OPLS-DA).

To determine the overall characteristics of the original data, the compound features were used for the principal component analysis (PCA). QC analyses were clustered closely in the PCA score plot, suggesting the good stability and reproducibility of the UHPLC-QE-MS-based compounds analysis. As shown in Figure 5A, the first two principal components accounted for 67.50% and 14.9% of the total variance, respectively. All samples were within the 95% confidence interval. The SBS samples and the control were classified into two groups: the SBS samples were grouped into the negative PC1, whereas the control was primarily positioned in the positive PC1. OPLS-DA, as a supervised recognition method, filtered out the noise irrelevant to the classification information and was used to establish a discriminant model [29] in order to highlight the relevant characteristics between the SBS samples and the control. The OPLS-DA model had high interpretability (R2X = 0.840; R2Y = 0.994) and predictability (Q2 = 0.976) (Figure 5B). No interaction was found between the two groups of samples with and without dried shrimp, and the distances between the data points were far apart. To prevent the overfitting of the model, 200 permutation tests were used for verification in this work. The results showed that the verification intercepts of R2 and Q2 were 0.82 and 0.02, respectively (Figure 5C), indicating the goodness of fit and predictive ability of the models without overfitting. These results indicated that the SBS samples were noticeably distinct from the control group, indicating that dried shrimp had a significant effect on the compounds (such as proteins, lipids, amino acids, carbohydrates, etc.) of sheep bone soup during the stewing process.

### 3.4. Identification and Content Analysis of Differential Taste Compounds

**Figure 6:**
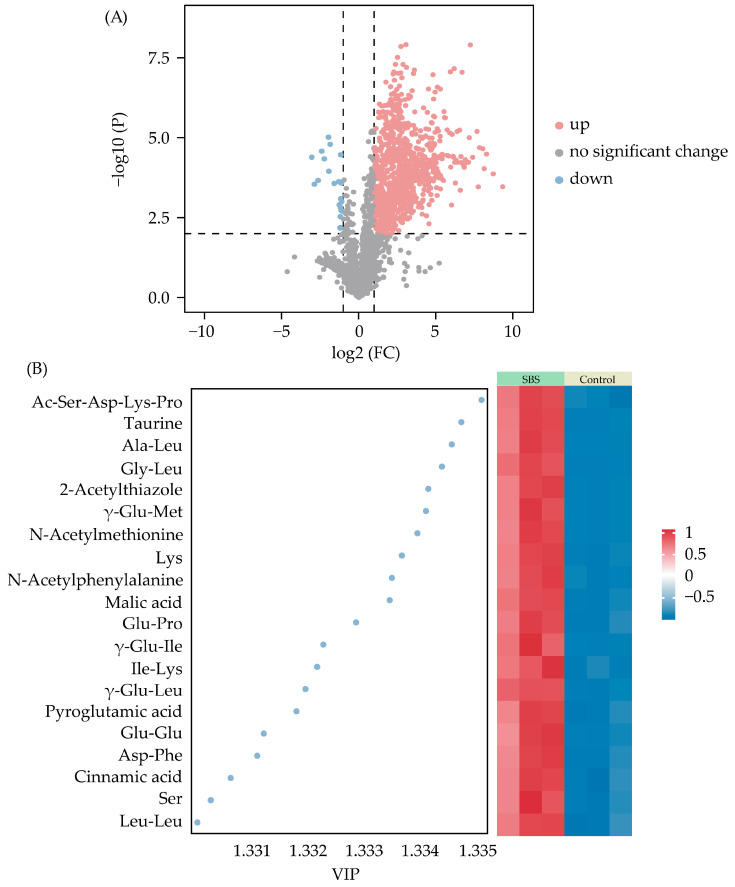
Screening results of differentially compounds ((**A**) volcano plot of differential compounds; (**B**) heat map of top 20 differential taste compounds in SBS samples and control).

Based on the results of OPLS-DA, parameters such as *p* < 0.01, fold changes (FCs) > 2 or FCs < 0.5, and VIP > 1 were used to screen for differential compounds in order to clarify the effect of dried shrimp addition on the flavor substances of SBS. As shown in Figure 6A, a total of 1212 differential compounds were screened, of which 1191 were upregulated and 21 were downregulated, mostly belonging to lipids and lipid-like molecules (458 compounds, 37.79%) and organic acids and their derivatives (251 compounds, 20.71%). In their previous study, Suleman et al. reported that lipids and lipid-like molecules are important components that affect the quality of sheep bone soup [7].

In addition, the analysis of the top 20 characteristic and differential taste compounds is shown in Figure 6B. The content of these differential taste compounds is shown in Table S4. After adding dried shrimp, the type and content of amino acids and peptides increased significantly. An obvious increase in two amino acids, Lys and Ser, both of which contribute to the sweetness of food, was detected in SBS [30]. Combined with soup samples, it was suspected that the taste profile characterized by well-balanced umami and sweetness flavors was related to the higher content of Lys and Ser. The content of ten dipeptides, including Ala-Leu, Gly-Leu, γ-Glu-Met, Glu-Pro, γ-Glu-Ile, Ile-Lys, γ-Glu-Leu, Glu-Glu, Asp-Phe, and Leu-Leu, was higher in the SBS samples than in the control. In a previous study, it was found that γ-glutamyl dipeptide compounds have a kokumi taste and enhance the umami intensity of soups [31,32]. In addition, Ala-Leu, Glu-Pro, Glu-Glu, and Asp-Phe contribute to umami taste [33,34,35,36]. Gly-Leu has similar bitterness values as free Leu, but Leu-Leu and Ile-Lys have a greater bitterness intensity because of the synergistic effects [37]. The content of taurine, a bitter-tasting compound, was also found to be 70 times higher in the SBS samples than in the control. While the bitter taste is easily perceived and lasts longer in the mouth, an ideal amount in SBS may help weaken the intensity of the sourness and improve the taste profile [38]. In addition, an increase in N-acetylmethionine and N-acetylphenylalanine was also found in SBS. They both showed that a better effect on kokumi enhancement [39]. The content of Ac-Ser-Asp-Lys-Pro significantly increased from 0.07 mg/L (control) to 1.08 mg/L (SBS). This peptide contains an umami-tasting amino acid (Asp) and some sweet-tasting amino acids (Ser, Lys, and Pro), supporting the idea that Ac-Ser-Asp-Lys-Pro contributes to taste. A previous report also indicated that the acetylation of the N-terminal amino group was effective in reducing bitterness intensity and enhancing umami intensity [40,41]. Pyroglutamic acid is the most abundant taste compound (umami) in soup samples, ranging from 111.23 to 1964.33 mg/L, and plays an important role in the overall taste of soup [42]. Cinnamic acid is a flavonoid that is found in cinnamon. When its concentration is increased, the perception of umami and sweetness is enhanced [43]. In addition, SBS is rich in malic acid, resulting in a more sour-tasting soup [44]. 2-acetylthiazole (with a popcorn-like aroma) was 85 times more abundant in the SBS samples than in the control, which may be attributed to the onset of fatty acid thermal oxidation [45]. Furthermore, these different taste compounds have been used as fatigue recovery agents, liver function enhancers, ammonia detoxification agents, and nutritional supplements in a variety of refreshing beverages [46,47].

## 4. Conclusions

In summary, the stewing process of sheep bone soup with dried shrimp was optimized, and the effect of dried shrimp on the flavor profiles of sheep bone soup was investigated from a chemical composition perspective. The optimal preparation process for the stewed sheep bone soup with dried shrimp was a content of 30% of dried shrimp added, a material–water ratio of 1:4.5, and a stewing time of 2.7 h. Under these optimal conditions, the aroma, umami, kokumi, and texture intensity of SBS increased significantly. The untargeted approach found that a total of 1212 differential taste compounds were screened, of which 1191 were upregulated and 21 were downregulated, mostly belonging to lipids and lipid-like molecules (458 compounds, 37.79%) and organic acids and their derivatives (251 compounds, 20.71%). The addition of dried shrimp significantly increased the content of taste compounds including sweet-tasting compounds (Lys and Ser), umami/umami-enhancing compounds (Ala-Leu, Glu-Pro, Glu-Glu, Asp-Phe, pyroglutamic acid, and cinnamic acid), bitter-tasting components (Gly-Leu, Leu-Leu, Ile-Lys, and taurine), kokumi-tasting compounds (γ-Glu-Met, γ-Glu-Leu, γ-Glu-Ile, N-acetylmethionine, and N-acetylphenylalanine), a sour-tasting compound (malic acid), and a popcorn-like aroma compound (2-acetylthiazole). In addition, an increase in Ac-Ser-Asp-Lys-Pro was also found and its contribution to improving the taste of soup cannot be overlooked. These findings reveal the contribution of dried shrimp to the improvement of the flavor of sheep bone soup and provide a theoretical basis for the processing of SBS. Furthermore, most of the differential taste compounds were proven to have physiological function activities, and further exploration of the differential taste compounds between SBS and the control will be helpful in the development of functional SBS products.

## Figures and Tables

**Table 1:** The results of the orthogonal test.

Group	Factors			Scores
	Material–Water Ratio (A)	Stewing Time (B)	Content of Dried Shrimp Addition (C)	
1	1	1	1	10.2
2	1	2	2	10.4
3	1	3	3	10.5
4	2	1	2	10.3
5	2	2	3	10.2
6	2	3	1	10.0
7	3	1	3	10.6
8	3	2	1	10.4
9	3	3	2	10.7
$\bar{K_{1}}$	31.1	31.1	30.6	
$\bar{K_{2}}$	30.5	31.0	31.4	
$\bar{K_{3}}$	31.7	31.2	31.3	
Range (R)	1.2	0.2	0.8	

## Data Availability

The original contributions presented in the study are included in the article/Supplementary Materials. Further inquiries can be directed to the corresponding author.

## Supplementary Materials

The following supporting information can be downloaded at: https://www.mdpi.com/article/10.3390/foods14081425/s1, Table S1: parameter settings of single-factor test; Table S2: parameter settings of orthogonal test; Table S3: sensory evaluation criteria; Table S4: the content of top 20 differential taste compounds identified by UHPLC-QE-MS.
