# Supplementary material for: Analyzing the Effect of Dried Shrimp on the Flavor of Sheep Bone Soup Through Sensory Evaluation Combined with Untargeted Approaches
Source: Foods. 2025 Apr 21;14(8):1425. doi: 10.3390/foods14081425 (PMC12026518; doi:10.3390/foods14081425)
Supplement: Supplementary file 1 [file foods-14-01425-s001.zip › foods-3529803-supplementary.pdf]

Table S1. Parameter settings of single-factor test

Group	Material-water ratio	Stewing time (h)	The content of dried shrimp addition (%)
1	1:4	2.0	10<br>20<br>30<br>40<br>50
2	1:4	1.0<br>1.5<br>2.0<br>2.5<br>3.0	30
3	1:2<br>1:3<br>1:4<br>1:5<br>1:6	2.5	30

Table S2. Parameter settings of orthogonal test

Level	Factor		
	Material-water ratio (A)	Stewing time (B)	The content of dried shrimp addition (C)
1	1:3.5	2.3	25
2	1:4.0	2.5	30
3	1:4.5	2.7	35

Table S3. Sensory evaluation criteria

Parameter	Standard	Score
Color	Dark and dull	1 ~ 4
	Yellow	5 ~ 7
	Milky	8 ~ 10
Aroma	Nondetectable	1 ~ 5
	Slight aroma with slight off-flavor	6 ~ 10
	Rich and pleasurable aroma without off-flavor	11 ~ 15
Umami	Nondetectable	1 ~ 5
	Clear but insufficient or heavy	6 ~ 10
	Rich, full, mild, well-balanced and pleasurable	11 ~ 15
Kokumi	Nondetectable	1 ~ 4
	Slightly thick	5 ~ 7
	Obviously thick	8 ~ 10
Texture	Thin and tasteless	1 ~ 4
	Slightly smooth, delicate and balancing	5 ~ 7
	Smooth, delicate and balancing	8 ~ 10
Overall impression	Much bad	1 ~ 4
	Acceptable	5 ~ 7
	Pretty good	8 ~ 10

Table S4. The content of top 20 differential taste compounds identified by UHPLC-QE-MS.

No.	Compounds	*Target* (*m/z*)	R_T (min)	Content (mg/L)		VIP	*P* value	Fold Change	Catergory
				SBS	Control				
1	Ac-Ser-Asp-Lys-Pro	486.22	3.96	1.08	0.07	1.33510	0.00003	15.30	Organic acids and derivatives
2	Taurine	124.01	0.94	319.91	4.57	1.33473	0.00004	70.06	Organic acids and derivatives
3	Ala-Leu	201.12	3.03	14.91	0.11	1.33456	0.00006	139.06	Organic acids and derivatives
4	Gly-Leu	187.11	3.02	61.81	0.73	1.33439	0.00001	84.19	Organic acids and derivatives
5	2-Acetylthiazole	126.00	0.91	14.60	0.17	1.33415	0.00006	84.92	Organic oxygen compounds
6	γ-Glu-Met	277.09	3.59	9.97	0.09	1.33410	0.00007	110.10	Organic acids and derivatives
7	N-Acetylmethionine	190.05	4.65	16.49	0.26	1.33395	0.00008	62.81	Organic acids and derivatives
8	Lys	145.10	0.76	26.44	0.94	1.33367	0.00005	28.14	Organic acids and derivatives
9	N-Acetylphenylalanine	206.08	5.72	9.55	0.32	1.33349	0.00007	29.72	Organic acids and derivatives
10	Malic acid	133.01	1.06	52.16	1.87	1.33346	0.00001	27.92	Organic acids and derivatives
11	Glu-Pro	225.09	3.88	33.04	1.83	1.33286	0.00006	18.07	Organic acids and derivatives
12	γ-Glu-Ile	259.13	3.50	47.74	0.39	1.33227	0.00005	120.96	Organic acids and derivatives
13	Ile-Lys	258.18	1.14	2.38	0.06	1.33216	0.00005	42.44	Organic acids and derivatives
14	γ-Glu-Leu	259.13	4.67	22.71	0.78	1.33195	0.00000	29.10	Organic acids and derivatives
15	Pyroglutamic acid	128.03	1.69	1964.33	111.23	1.33179	0.00011	17.66	Organic acids and derivatives
16	Glu-Glu	257.08	1.69	58.94	2.35	1.33121	0.00019	25.10	Organic acids and derivatives
17	Asp-Phe	279.10	4.43	2.75	0.15	1.33109	0.00013	18.46	Organic acids and derivatives
18	Cinnamic acid	147.04	3.61	18.10	1.74	1.33062	0.00012	10.38	Phenylpropanoids and polyketides
19	Ser	104.03	0.88	4.95	0.38	1.33026	0.00014	13.03	Organic acids and derivatives
20	Leu-Leu	243.17	4.43	3.66	0.29	1.33002	0.00007	12.66	Organic acids and derivatives
